# The Influence of Four Constructs of Social Support on Pregnancy Experiences in Group Prenatal Care

**DOI:** 10.1089/whr.2020.0113

**Published:** 2021-06-02

**Authors:** Kalyn M. Renbarger, Jean Marie Place, Melanie Schreiner

**Affiliations:** ^1^ School of Nursing, Ball State University, Muncie, Indiana, USA.; ^2^ Department of Nutrition and Health Science, Ball State University, Muncie, Indiana, USA.; ^3^ IU Health Ball Memorial Family Medicine Residency Center, Muncie, Indiana, USA.

**Keywords:** group prenatal care, social support, pregnancy, experiences

## Abstract

***Objective:*** This study aimed to identify the influence of the four constructs of social support on positive pregnancy experiences in CenteringPregnancy, a group prenatal care (GPNC) model.

***Methods:*** Using a qualitative descriptive design, semi-structured interviews were conducted with 11 women who had participated in at least 6 of 10 GPNC sessions at a family practice medicine residency. Participants were asked to describe their experiences in GPNC.

***Results:*** Using a standard content analysis, four constructs of social support (emotional, informational, instrumental, and appraisal) were identified through three major themes: (1) informational support, offered by peers in GPNC settings, promotes learning and prepares women for motherhood; (2) emotional and appraisal support, offered by peers in GPNC, improves emotional well-being and helps women build lasting, supportive connections with peers, and (3) emotional, informational, instrumental, and appraisal support work in tandem to create positive relationships between women and health care providers.

***Conclusion:*** Social support provided a means to a positive prenatal health care experience that facilitated the attainment of new knowledge and the formation of positive relationships with health care providers and peers. The findings of this study can provide health care providers with a framework to examine and enhance their practice and care of women in the perinatal period.

## Introduction

Pregnancy and the transition to motherhood is a period of profound change for many women. As such, social support is a critical protective factor for women undergoing the physical and emotional changes of the perinatal period.^1^ Social support is linked to improvements in maternal quality of life, higher rates of continued breastfeeding, and breastfeeding self-efficacy.^2–4^ Adequate social support promotes mental health in the perinatal period by reducing low mood and anxiety and helping women manage feelings of isolation and disempowerment.^1^ As women transition to motherhood, social support also enhances maternal competence by providing encouragement, promoting self-esteem, and decreasing stress.^1,5^

Conversely, low levels of social support in pregnancy have been associated with poor maternal and infant health outcomes.^6^ Women with low social support were found to have infants with the highest levels of cephalization, indicating higher levels of asymmetric fetal growth.^7^ Women who report inadequate social support during pregnancy may be more likely to experience depression and anxiety after the birth of their infant.^8,9^ A study discovered that both adolescent and adult women with low levels of social support were approximately five times more likely to experience postpartum depression.^10^

Traditional models of prenatal care in the United States, where women receive individual health care services from clinical providers during pregnancy such as education, physical assessments, and guidance on preventing obstetric complications, can be important sources of support and help promote positive health outcomes for women and infants. Some women, however, have voiced concerns with traditional models of prenatal care, citing frustration with long wait times for new patients, fragmented care, unresponsive staff, and poor communication with providers.^11,12^ Women have also reported health care providers who ignored their concerns, did not spend adequate time during appointments to address their concerns, and made them feel unwelcomed during prenatal care.^11^ Negative prenatal care experiences act as barriers to meeting the complex needs of pregnant women.^11–13^ Consequently, women may not receive the support they need during pregnancy, potentially contributing to negative health outcomes.

As an alternative to traditional models of prenatal care, group prenatal care (GPNC), where women receive care in small groups and participate in group discussion covering a variety of health topics related to pregnancy, has been shown to boost women's perceptions of social support.^2,14^ CenteringPregnancy, an evidence-based GPNC model, provides prenatal care through a supportive group process.^15^ Health care providers facilitate discussion and engage in interactive learning activities with group members (*i.e.*, both pregnant women and support people such as spouses or partners) during CenteringPregnancy. Providers also oversee and encourage women to conduct selected self-examinations, like taking blood pressure, upon arrival to the group sessions. Time is also reserved during the group sessions for providers to conduct brief, one-on-one, private physical assessments with women. The providers are tasked with guiding discussions based on prechosen topics and at times must counter any erroneous information presented by other members of the group. If the CenteringPregnancy providers feel information discussed by a group member was not accurate, they will provide factual information.

Women participating in CenteringPregnancy have indicated they felt safe in sharing information and expressing fears, questions, concerns, and experiences.^14^ Importantly, women identified social support as a crucial component that underpinned their satisfaction with CenteringPregnancy GPNC.^14,16^

The conceptual framework of this study is based on the work of House^17^ where social support has been defined as an individual's perception and actuality that one is cared for and has available assistance from others, and that an individual is part of a supportive social network.^17,18^ Social support consists of four functional constructs identified as emotional, instrumental, informational, and appraisal.^17,19^ Emotional support has been described as expressions of empathy, love, trust, and caring and can be offered through gestures of affection. Informational support is the provision of valuable advice, suggestions, and information to assist an individual in solving problems. Instrumental support is tangible assistance and may include giving financial support or a type of service to the individual. Informational and instrumental support are sometimes included in the same social support category.^20^ Finally, appraisal support is an accurate assessment and constructive feedback of the current situation provided to an individual for self-evaluation, affirmation, and encouragement.^17,19^ These four constructs of social support may present a “buffering effect” where resources provided to an individual may enhance health benefits and decrease the negative consequences of stressors experienced by an individual.^19,21,22^ Adequate social support promotes healthy coping mechanisms when individuals experience stressors or a major life change such as the birth of a child.^19,23^

While studies have identified social support as an important component to GPNC, to our knowledge, no study has explored the different ways in which social support contributes to positive health care experiences. Therefore, the purpose of this article is to identify and describe the constructs of social support in GPNC settings that promote positive health care experiences among women in the perinatal period. The findings of this study can enhance how health care providers deliver GPNC by identifying the ways in which different constructs of social support meet the unique needs of women in the perinatal period and recognizing the ways in which providers and peers can positively contribute to women's prenatal care experiences.

## Materials and Methods

### Design

A qualitative descriptive design was used in this study to produce a narrative summary to describe social support in GPNC based on the participants' perspectives.^24^ This approach provides descriptions of narrative data that can be used to provide information needed by health care providers to address health care concerns.^24^ A qualitative descriptive design was chosen because semistructured interviews allow participants to speak freely about their experience in GPNC and produce findings that are minimally theorized. The reporting of this study was guided by the Consolidated Criteria for Reporting Qualitative Research using a checklist of 32 criteria to enhance the reporting of interview and focus group studies.^25^

### Recruitment

A purposeful sample of women was recruited from a family medicine residency center in the Midwestern region of the United States that offered CenteringPregnancy GPNC as an option women could choose to enroll in at their initial obstetric appointment. The residency center is well known for providing health care services to underserved and low-income clients. In the CenteringPregnancy model at the family medicine residency center, women were scheduled for 10 group prenatal visits of 120 minutes each, in addition to the initial prenatal examination and visit with their primary care physician. Participants were eligible to participate in this study if they attended at least 6 of the 10 sessions of the CenteringPregnancy prenatal care schedule. After delivery, women who were involved in the group sessions were invited to participate in an informal, celebratory gathering to celebrate the births of their children. During individual phone calls before the event, a nurse notified women of the opportunity to participate in the qualitative interview. If a woman agreed to be contacted, the second author who was present at the celebratory gathering invited the woman to be interviewed in a separate, private space. The nurse, as well as the second author, was familiar to the women since both had, at times, been present at the group sessions with the women. Participants were given a written informed consent document and the participants had adequate time to read the document and ask questions before beginning the interview. All interviews were conducted after participants signed informed consent and gave verbal permission to be recorded. The study was approved by the center's Institutional Review Board.

### Data collection

Data collection took place over the course of four cohorts of CenteringPregnancy GPNC sessions between June 2015 and September 2017 and consisted of one-time, one-on-one semistructured interviews with open-ended questions. In the interview guide, participants were asked about their experience in CenteringPregnancy, as well as what they liked and did not like. We took this general approach, consistent with qualitative description design, to listen for the words participants used in describing their experience, particularly as it related to their experience feeling supported or not in the GPNC model. Finally, we asked about whether and how the participants felt their experience in the group sessions prepared them for the realities of taking care of their new child. Participants filled out a brief demographic questionnaire following the interview. Participants were assigned study codes to facilitate linking the qualitative interviews with the demographic data. The interviews were designed to last 15–30 minutes and the average interview lasted ∼15 minutes.

### Data analysis

Interviews were audio-recorded and transcribed by a professional transcriptionist. The data were analyzed by the first and second authors with expertise in qualitative research and maternal and child health. A standard content analysis was conducted using steps outlined by Miles et al.^26^ First, the first and second authors read through all transcripts to obtain a comprehensive understanding of the nature of data. Second, the first and second authors concluded that social support was a dominant theme throughout all the transcripts. Third, the first and second authors highlighted and extracted each text unit related to social support in a GPNC setting. Each text unit was assigned a code that described the nature of the participants' statements. The codes were verified by another team member. The codes were then reviewed by the research team and then categorized according to the type of social support received by the participant as described in House's^17^ social support theory. Finally, the first author wrote a narrative description of the final categories, which was reviewed by the second and third authors.

Trustworthiness and credibility were enhanced through peer debriefing among the team members, which occurred at regular intervals throughout the data analysis process. All data analysis decisions were recorded through a detailed audit trail maintained by the first and second authors.

## Results

The sample included 11 women from the residency center. The women were between the ages of 19 and 38 years (out of 8 participants who reported their age). The majority of the participants (*n* = 8) had not experienced a prior pregnancy that resulted in a living birth and were not currently parenting another child. Three women reported prior live births and were parenting other children. Four participants reported their current pregnancy as unplanned. All participants (*n* = 11) delivered their children in an urban hospital setting. Demographic information is displayed in Table 1.

**Table 1.** Demographic Characteristics

Demographic characteristics	Participants
Age	19–38 years (*n* = 8) Not reported (*n* = 3)
Race/Ethnicity	Non-Hispanic/White (*n* = 2) Biracial (*n* = 1) Hispanic (*n* = 1) Not reported (*n* = 7)
Labor/delivery setting	Urban (*n* = 11) Rural (*n* = 0)
Prior living births	No prior living births (*n* = 8) Prior living births (*n* = 3)
Parenting other children	Yes (*n* = 3) No (*n* = 8)

Three themes for how social support contributed to positive pregnancy experiences among participants in GPNC were identified by the researchers. All four constructs of social support (emotional, informational, instrumental, and appraisal) were identified by participants in the three major themes: (1) informational support, offered by peers in GPNC settings, promotes learning and prepares women for motherhood; (2) emotional and appraisal support, offered by peers in GPNC, improves emotional well-being and helps women build lasting, supportive connections with peers; and (3) emotional, informational, instrumental, and appraisal support work in tandem to create positive relationships between women and health care providers.

It is important to note that participants mostly spoke positively about their interactions and the social support provided by their peers and health care providers in GPNC. On a few occasions, some participants were disappointed by the lack of participation when other women were unable to attend the group sessions. A few participants also indicated that one participant may have felt “left out” for being further along in gestation compared to the other women in the group.

### Informational support, offered by peers in GPNC settings, promotes learning and prepares women for motherhood

All the participants provided descriptions of the informational support that helped prepare them for motherhood. Informational support was achieved through an interactive learning process where fellow women in GPNC exchanged information, stories, questions, opinions, and advice. Informational support that was freely shared and gratefully received among group members contributed to a learning environment where participants were comfortable sharing personal and medical anecdotes with their peers. Most of the participants were able to describe instances where hearing another woman's stories facilitated their learning and helped them prepare for labor and delivery and different aspects of mothering. The act of sharing stories in a conversational, fun, and frank way was described by a participant as a “helpful” way to learn information. One participant described how the exchange of stories with other women helped her feel comfortable speaking about her diabetes and seek ways to appropriately manage her condition. Other participants indicated that information shared by women through the vehicle of a story was more valuable than hearing information from a health care provider in traditional prenatal care. One participant (p1) stated,

You're in there and you get more education, and you're with other girls, to hear about other girls and how they're feeling and there are other stories, and it's just, I feel like, more helpful than just hearing from a doctor that possibly tells the next pregnant woman the same thing, you know.

Another participant (p2) enjoyed providing informational support directly by answering other women's questions and stated, “Somebody that's younger may have a question that maybe we [other group members] can answer, and the doctors don't even have to, you know, so that's kind of fun.”

Most of the participants stated that they valued the advice and opinions of other women in the group, at times more than a doctor. These participants reported not feeling judged and were comfortable when asking other women for advice. In addition, these participants felt they received more personalized information on topics such as breastfeeding, labor and delivery, and morning sickness from other women who had experience with those topics. All the participants suggested the informational support received increased their knowledge and confidence as they transitioned to motherhood.

Three participants, who had previous pregnancy and parenting experiences, were hesitant to begin GPNC initially, but were able to gain new knowledge and “learn things they did not know before” from the informational support provided by peers in GPNC. One participant with a previous pregnancy and parenting experience (p3) described how being with peers helped her prepare for labor and delivery. She recalled, “They [other women] think about things that maybe you wouldn't think about-until you're in the delivery room, and then there's no time to think about it.” Because of their previous pregnancy and parenting experiences, these participants were sometimes referred to for information and advice by the other women in the group without such experiences. One participant (p9) who was pregnant for the first time recalled seeking their advice and stated, “And like if you had a question, like [group member's name] had breastfed before.”

### Emotional and appraisal support, offered by peers in GPNC settings, improves emotional well-being and helps women build lasting, supportive connections with peers

All the participants provided descriptions of emotional support that promoted positive peer relationships. Emotional support was facilitated when women shared their personal pregnancy experiences, listened to other women, and responded empathetically. Participants bonded over their shared pregnancy experience and enjoyed participating in GPNC with women who were expected to give birth around the same time period. Participants indicated that because the other women were experiencing pregnancy and motherhood at the same time, they felt an affinity to other women and perceived them as more understanding. One participant (p6) stated,

They [other group members] were really understanding, like if you didn't know something about babies or whatever, they [other group members] didn't make you feel like you were like stupid, or whatever. So they [other group members] were really understanding, which was helpful.

Another participant expressed that she felt “like you're at home” when attending GPNC and referred to other group members as her “friends.” Another participant (p11) elaborated on how support from other women during the GPNC sessions helped her cope with anxiety. She stated,

I think, well for me, being pregnant and being stuck at home all the time, I was able to get out of the house, you know, and come here and see people that I'm becoming friends with, because I have like severe anxiety disorder.

The benefits of emotional support from other women were enhanced when participants felt listened to and were provided empathetic responses. All the participants described feeling accepted, encouraged, cared for by other group members, and “like you're not really alone.” One participant described how she could “gripe about something” with other group members because she felt they would understand. This group of participants valued being heard and being able to voice their concerns in GPNC. One participant described how she valued being heard and receiving input from others. She stated, “Just them [other group members] talking and them [other group members] asking for your input on everything, instead of just kind of overriding you.” Several participants valued the emotional support from their peers and extended their relationships beyond the GPNC setting. One participant (p3) stated,

The coolest thing for me is that I've built friendships off of it. And a couple of the girls and I, we talk on *Facebook* and stuff still, and I went over and seen the babies, we talk about problems we're having.

Some participants of this study described how appraisal support assisted in building relationships with their peers. These participants described how their peers reduced their fears through the normalization of pregnancy. One participant stated (p9), “What means the most, is like you can share your fears, you can share everything, and we all became really close.” Most participants spoke of appraisal support more generally and appreciated being in a group setting with other women who were experiencing the same pregnancy-related changes. One participant stated (p10), “the girls that are there with you, they're going through the exact thing at the same exact time.”

### Emotional, informational, instrumental, and appraisal support work in tandem to create positive relationships between women and health care providers

All the participants described emotional, informational, instrumental, and appraisal support in GPNC that assisted them in building positive relationships with health care providers. Emotional support was provided by the health care providers through caring gestures and concern for the participants, while appraisal, instrumental, and informational support were described as enhanced in GPNC compared to traditional prenatal care.

Participants in the GPNC were more comfortable speaking openly with health care providers as the result of support provided by their peers in the group. Consequently, participants were able to develop a connection with health care providers, from which they received emotional support. One participant stated (p10),

You know, because that one-on-one is kind of awkward. You don't really state all your problems, because you don't really want to bother the doctor with it. But when you have other people that's going along the ride with you, you're more prone to be open, and I think that's what happened.

Participants described positive emotional connections with their health care providers in GPNC. One participant stated (p9), “Yeah, the way they interacted, the way the doctors interacted with us. I honestly think the residents, and [the doctor], and [nurse's name], they're more like family too because you can sit there and you can talk to them.” Another participant referred to speaking with health care providers as “more like talking to a shrink.” Several participants appreciated the emotional support and concern offered from their health care providers in GPNC that extended beyond the group setting. One participant (p1) stated, “But the [health care providers] are not just distant, they're not just here to do a class and then be done with you.” Another participant (p7) stated, “I liked her [health care provider]. She would call me, she would check on me and the babies, and I really felt, I liked that.” The exchange of personal information during group sessions facilitated emotional support and relationships with health care providers. One participant (p9) recalled a meaningful example of emotional support provided by a health care provider in GPNC. She stated, “When my mom died, [the doctor] sent me a card. She [the doctor] lost her mom I believe when she was eight, so she understood the feeling of losing your mom, and not many people understand that feeling.”

Three of the participants with previous prenatal experiences in traditional models of care preferred the appraisal, informational, and instrumental support received in GPNC compared to traditional prenatal care. GPNC facilitated this support by the amount of time participants spent with health care providers and the continuity of care available beyond the scheduled group sessions.

Compared to traditional prenatal care models, the GPNC setting facilitated relationships with health care providers by allowing participants more time with providers to make more personal connections. The time spent with providers in GPNC allowed participants to be more comfortable seeking needed appraisal support and encouragement outside the regularly scheduled GPNC sessions. One participant stated (p3),

I was able to call and say, hey, this is going on, I'm kind of freaking out, and she was like okay, this has happened so far, and these changes are normal, and her being readily available was definitely really nice.

Another participant (p1) who did not have prior prenatal care experience echoed receiving appraisal support from health care providers in GPNC and stated, “so just knowing that my sickness was not normal, but it was still okay, and they [providers] talked to me and told me about it and stuff like that.”

The three participants with previous prenatal experiences in traditional models of care preferred informational and instrumental support offered in GPNC. These participants believed the informational support received was the result of the amount of time spent with health care providers. One participant stated (p3), “Yeah, and in a regular doctor's appointment you're only in there for like 15 or 20 minutes and then you're leaving, so their [GPNC providers] information is a little better for a new parent.” These participants valued the continuity of care and perceived informational and instrumental support as more accessible in GPNC. They implied that their health care providers in GPNC were more responsive and did not make them “wait a day” to receive needed information or advice. These participants also described resources, offered by health care providers, such as books, articles, and other educational material as more “helpful” than what they received in traditional models of prenatal care and enhanced their experience. One participant stated (p1), “And with your one-on-one, if you go just to your doctor, you don't get those papers that you can go back through,” referring to extensive manuals on pregnancy that CenteringPregnancy participants receive. Another participant (p7) felt snacks offered by the health care providers enhanced her GPNC experience. She stated, “And you actually get to eat snacks, because being pregnant.” Consequently, the emotional, informational, instrumental, and appraisal support provided by health care providers in GPNC facilitated positive connections between the participants and their providers.

## Discussion

A central finding of our study was that the prenatal care experience was enhanced by four constructs of social support: (1) emotional; (2) informational; (3) instrumental; and (4) appraisal. In GPNC, women received social support from both health care providers and their peers. The mechanisms of social support allowed for a positive prenatal care experience through the attainment of new knowledge, building of positive relationships with health care providers, and forming supportive peer relationships.

The findings of this study suggest that GPNC offers a significant source of peer support, strengthens relationships with health care providers, and aids women in the transition to motherhood. Furthermore, our qualitative data indicate that peer support, channeled through relationships with other women in GPNC, is perceived to offer additional benefits of support over traditional models of care.

Previous studies have identified that GPNC can facilitate positive relationships with peers and health care providers.^27–29^ Participants in our study shared and received emotional support and were able to develop supportive relationships with others in their group. Several participants attributed their relationships during GPNC to decreasing their levels of anxiety. This finding supports prior studies that indicate women with adequate social support during the perinatal period report have decreased levels of anxiety and depression, have lower levels of the stress hormone cortisol, and have improved their self-esteem and ability to parent.^1,8,30^ In other research, peer support interventions are shown to increase women's attendance in local parent groups, increase women's confidence by normalizing parenting concerns, and increase self-efficacy to face challenges^1,31^

Emotional support received from health care providers is a crucial finding because frequently women report negative relationships with their providers in traditional models of prenatal care, particularly when they perceive their health care providers as judgmental, intimidating, or lacking empathy.^32–35^ Negative experiences with health care providers can serve as a barrier to receiving adequate care during pregnancy.^1^ GPNC can facilitate emotional support through supportive relationships with peers and health care providers to improve maternal health outcomes.

GPNC may be key in promoting *informational* and instrumental support for women during the perinatal period. Participants reported gaining knowledge to solve problems—a type of informational support—which helped prepare them for motherhood. This echoes prior research that suggests information sharing through social support networks can increase labor confidence and parenting competence.^1,36–38^ Other studies have found that the GPNC setting facilitates health education and supports maternal child health outcomes.^31,39^ This finding is noteworthy because women often report they do not receive the health information they need during pregnancy.^40–42^ The informational support provided in GPNC may provide a means to adequately educate women during pregnancy with personalized information they are seeking. It is essential to consider that women do not always receive accurate information from peers during the perinatal period,^43^ which highlights the significance of the health care provider's role during GPNC.

Participants reported preferring both informational and instrumental support from health care providers in GPNC over that offered in traditional prenatal care models. Oftentimes, it was the delivery of the support that facilitated relationships with the health care providers in GPNC. Other studies confirm that women tend to perceive more attention from health care providers, more efficient care, and more responsive care in GPNC.^29,44^ Consequently, women participating in GPNC have reported increased satisfaction with care, attended prenatal appointments more often, and were more likely to participate in health promoting behaviors compared to women who attend traditional prenatal care.^39,45^ This finding is noteworthy because satisfaction with prenatal care and health care providers may facilitate health care utilization.^46^

GPNC may enhance appraisal support for women in the perinatal period. Participants reported receiving appraisal support from health care providers and peers during GPNC. Participants appreciated the normalizing of their pregnancy by both the health care providers and peers. Women were comfortable discussing concerns they had because they believed other women were experiencing similar issues and they appreciated having their pregnancy-related issues affirmed by the health care providers. Other studies have found that normalizing pregnancy reduced women fears about pregnancy-related changes and labor and delivery.^28,29,47^ Appraisal support during pregnancy is important in building maternal confidence. Appraisal support has been shown to increase confidence in infant care.^37^

The limitations of this study should be considered. First, recruitment ended with a small sample size of 11 participants due to limited grant funding. The small sample size limited the scope of findings by reducing the descriptions of social support in prenatal care that could be analyzed and coded. It is possible that individuals who were unavailable for interviews may have had different experiences in GPNC. Although qualitative studies are generally purposive, more studies need to be conducted using a larger sample size to allow the collecting of more descriptions of social support in GPNC. In addition, interviews with participants were held during a celebratory event; the nature of the celebratory event and the fact that some of the participants brought their infants to the interviews may have impacted the length and depth of the interviews, given that some participants may have wanted to quickly return to the festivities. Next, demographic data were not consistently reported by all the participants. Therefore, we are unable to determine how demographic factors such as race, culture, and ethnicity may influence the findings of this study. Future studies should be conducted to include diverse populations to determine how social support in GPNC may be perceived by different race/culture/ethnic groups. Another limitation to this study is selection bias. It is possible that women who attended fewer than six sessions may have had a less positive experience and less supportive interactions. We did not consider the length of program exposure on the findings of this study. Finally, participants were asked to recall their experiences in GPNC during the interview. Some participants may not have been able to recall some details due to the lapses in time and memory. However, most participants were able to provide robust details of social support when describing their experiences in GPNC.

## Conclusion

Social support benefits women in GPNC settings by helping to create a positive prenatal experience through the four constructs of emotional, informational, instrumental, and appraisal support. The study's findings can provide health care providers with a framework to examine and enhance their practice with women in the perinatal period. Health care providers working in a variety of settings should consider the benefits of social support when caring for women during pregnancy. Health care providers are in a key position to assess women's social support and offer them resources, including the GPNC model to assist in mobilizing support during pregnancy. Maternal child health outcomes may be improved through social support in GPNC by helping women make meaningful social connections, enhancing relationships with health care providers, and providing a supportive learning environment.

## Abbreviation Used

GPNC: group prenatal care
